# The unmet needs of older adults living in nursing homes in Mainland China: a nation-wide observational study

**DOI:** 10.1186/s12877-022-03699-9

**Published:** 2022-12-22

**Authors:** Deqin Huang, Tieying Zeng, Jing Mao, Meizhen Zhao, Meiliyang Wu

**Affiliations:** 1 Department of Nursing, Tongji Hospital, Tongji Medical College, Huazhong University of Science and Technology, 1095 Jiefang Avenue, 430030 Wuhan, Hubei Province China; 2 School of Nursing, Tongji Medical College, Huazhong University of Science and Technology, 13 Hangkong Road, Qiaokou District, 430030 Wuhan, Hubei Province China

**Keywords:** Unmet needs, Met needs, Older adults, Nursing home, CANE

## Abstract

**Background:**

The unmet needs of older adults in nursing homes could result in their poor health status physically and psychologically. The aim of this study was to understand the characteristics of unmet needs of older adults residing in nursing homes in China, and to probe into the contributing factors.

**Methods:**

In this cross-sectional design, the demographic and health status questionnaire, Modified Barthel Index, the Numerical Rating Scale for pain assessment, Geriatric Depression Scale, Camberwell Assessment of Need for the elderly were employed to survey older adults living in 38 nursing homes in 13 cities in China from July 2017 to June 2018 through a multi-stage, stratified sampling scheme. The Short Portable Mental Status Questionnaire was adopted to exclude participants with severe cognitive impairment. Aside from descriptive analysis, a raft of hierarchical logistic regression models were run by sequentially controlling for the independent variables at 5 levels (demographic characteristics, health status, pain, ADL, and depression), aiming to identify the influencing factors of the unmet needs of the residents.

**Results:**

The effective sample size involved 2063 older adults (63.4% female versus 36.6% male), with a response rate of 98.5%. The median and inter-quartile range of the total needs and unmet needs of the sample was 3(1, 4) and 0(0, 1) respectively, with 122 older participants having more than 3 unmet needs (high unmet need category) versus 1922 older ones having ≤ 3 unmet needs (low unmet need category). The unmet needs of older adults in nursing homes mainly fell into social domains. Gender, religion, educational background, marital status, living condition before admission, room type, incomes, staffing, number of diseases, pain, Barthel Index, and depression were contributive to unmet needs of older adults in long-term care facilities in the final model that was adjusted for all levels of variables (all *p* < 0.05).

**Conclusion:**

Understanding the influencing factors of the unmet needs of older adults in long term care provides clues for healthcare professionals to offer better care for this population. System-level support to nursing homes and training of staff are highlighted. Plus, taking measures to beef up social connections for the older adults to meet their social needs was suggested.

## Background

By October 2020, the world’s elderly population over the age of 65 has reached 728 million, with the number expected to double by 2050 [1]. According to statistics, as of May 2021, the population aged 65 and above hit 190.64 million, occupying 13.5% of all population [2]. The trend of having so many people getting old will only spiral up in the coming decades. With a grey wave washing over China, pressure on government pension plans mounts. Meanwhile, shrinking family size as a result of decades long one-child policy makes families ill prepared for ageing population. Adding to the woes, expensive housing and meager pay has made physically impossible the old community norms of older adults living with several generations under one roof and relying on children for support. Families have been increasingly unable to meet the growing needs of geriatric care. Therefore, a growing number of older adults choose to live in nursing homes [3].

Older adults living in nursing homes have physical, psychological, social and environmental needs. Meeting the needs of residents are at the heart of nursing home service. Issues concerning whether and how residents’ needs are met are directly related to individual health outcomes [4], and have received abundant attention from stakeholders, especially from families and residents themselves.

Compared with older adults with no needs, met need or unclear needs, the unmet needs of them in nursing homes should be more concerned by medical staff [5]. Unmet needs refer to the fact that the problems of the older adults in nursing homes have not been solved by medical care services, resources, etc., and individuals have not received appropriate assessment and intervention to meet their needs [6, 7]. Unmet needs are important clinical and research indicators that can lead to lower quality of life, more anxiety and depression, and even higher mortality [4]. Thus, unmet needs are better predictors for negative outcomes than common measures such as met needs. Only by knowing what needs of the older adults in nursing homes have not been met, will we be able to take targeted measures tailored to them, thus truly improving the quality of life of them and other health outcomes.

There have been several studies probing into the unmet needs of nursing home residents using CANE, such as researchers from Poland, Netherlands, etc. [6–8]. However, evidence from China, a country with the largest share of elderly population on earth, has not been found. A clear understanding of the unmet needs of Chinese older adults in nursing homes is needed to compare with previous studies and to provide clues for medical staff to take countermeasures to meet the unmet needs of older adults in nursing homes and improve their health outcomes. Hence, the aim of this study was to use CANE to understand the needs of Chinese older adults in nursing homes, especially the situation and the predictive factors of unmet needs.

## Methods

### Data collection

In this study, we select older adults living in nursing home through a multi-stage, stratified sampling scheme. Firstly, per geographical divisions of mainland China by the Statistical Yearbook of Chinese Health and Family Planning, 2 provinces / municipalities from each of the Eastern, Central and Western Regions of China were randomly picked, totaling 6 provinces (Liaoning, Hainan, Shanxi, Hubei, Sichuan and Shaanxi). Then, 2 cities from each province were conveniently picked with consideration of travel accessibility. Meanwhile we added 6 back-up cities conveniently, forming a total of 18 cities. Thirdly, 3 nursing homes of various sizes (one having < 100 beds, one having 100–300 beds, and one having > 300 beds, respectively deemed as small size, medium size, and large size according to the official website of the Civil Affairs Bureau) in each city were reached out conveniently, totaling 54 nursing homes. Despite our desperate effort, 38.7% selected nursing homes declined our request for survey. In worst case scenario, we failed to obtain permissions from any of the selected nursing homes in 2 provinces (Liaoning and Shanxi). Therefore, we made adjustment to the sampling scheme by switching to other provinces/ municipalities and adding more cities to fill the vacancy. Finally, 38 nursing homes in 13 cities granted permission and were approached for the cross-sectional questionnaire survey which stretched from September 2017 through June 2018. Inclusion criteria of respondents were: 1) ≥ 60 years old [2]; 2) living in nursing homes for more than 1 month; 3) being consented for the survey. The exclusion criteria were: (1) the score of the Short Portable Mental Status Questionnaire for the older adults was less than 5 points; (2) having severe impairment in communication [4]. We used the self-made demographic and health status questionnaire, the Chinese version of CANE revised by expert consultation, Modified Barthel Index [9], The Numerical Rating Scale for pain assessment (NRS for pain assessment) [10] and the 15-item Geriatric Depression Scale [11] to survey the basic information and health status, needs, activities of daily living (ADL), pain, and depression of the older adults living nursing homes, respectively. The questionnaires were filled out through face-to-face interviews with the respondents by 12 nursing graduate students. Before the survey, the students were given unified training on surveying technique and countermeasures to problems emerging during the survey. The interviews took place in private places such as lounges or the participants’ rooms to minimize distractions and insure the authenticity of the data.

The sample size was calculated according to the formula *n* = Z_α/2_^2^×p×(1-p)/δ^2^ [12], where α = 0.05, Z_α/2_=1.96, and δ = 2.5%. Based on the survey results of the Ministry of Civil Affairs of China, the occupancy rate of nursing homes in 2020 was 50% [13]. On the ground of an 85% effective response rate, the sample size of this study was 1808. A total of 2094 questionnaires were sent out and 2063 were effectively received with a response rate of 98.5%.

### Measurements

#### Camberwell Assessment of Need for the Elderly (CANE)

Camberwell Assessment of Need for the Elderly (CANE) [14] is a measuring tool that can comprehensively assess the physical, psychological, social and environmental needs of older adults. This questionnaire was developed by Reynolds in 2000. It was first used to survey the needs of older patients with mental disorder, and was later expanded to general older population [15]. CANE consists of three versions, intended respectively for older population, home caregivers, and health care providers. This study chose the older adults version. CANE questionnaire looks into whether the needs of older adults are met or not, and groups them into either of the 4 categories of no needs, met needs, unmet needs, and unclear needs [14].

This questionnaire enjoys good reliability and validity, and can comprehensively assess the needs of older adults [4], which has been widely used in foreign countries. The 24-item questionnaire included social, physical, psychological and environmental needs. Each item addresses a specific need, with the response option 0 meaning there is no need, 1 meaning the need is met, 2 meaning the need is not met, and 9 meaning the need is not clear. The survey result of the questionnaire item is presented with the count of 4 categories (no need, met needs, unmet needs and unclear needs), without using its scoring values. The number of total needs is the tally of met needs plus the tally of unmet needs.

After obtaining the authorization of the original author, the research team translated CANE into Chinese, and then invited graduate students majoring in English to back translate the initial Chinese version. After confirming that there was no deviation and ambiguity caused by translation, expert consultation method was used to test the content validity of the Chinese version. Twelve experts (5 males and 7 females) involved in the consultation included managers of nursing homes, managers of hospital geriatric nursing, college researchers on older population. These experts came from Hubei Province and were aged 40 to 57 years old (median: 48 years old). Seven experts had a senior title, 3 had a deputy senior title, and 2 intermediate title (these two were presidents of nursing homes). The expert consultation form adopts a 4-level scoring method: experts give scores as specified by the correlation between the questionnaire items and the research content, 4 meaning very relevant, 3 relevant, 2 general, and 1 irrelevant. The Item-level content validity index (*I-CVI*) was obtained by dividing the number of experts giving 4 or 3 for each item by the total number of experts participating in the consultation. There is also a comment column in the consultation form for experts to give suggestions on adding, deleting or modifying the items in the tool.

The *I-CVI* of Chinese version CANE ranged from 0.75 to 1, and the average *S-CVI* was 0.93, indicating the tool enjoys good content validity. The authority coefficient of the experts ranged from 0.77 to 0.97, signifying that the consultation results were accurate and credible. After consulting with the experts, the researchers deleted item 5 (taking care of others), 20 (drinking), and 23 (paying your own bills). An initial sample of 201 respondents from 4 nursing homes in Wuhan city participated in the pilot survey from July to August 2017, in which, item 1 (accommodation) was deleted as all participants rated the need as “met”. At last, 20 items covering diet, self-care and daily activities were retained. The Cronbach’s alpha coefficient of the Chinese version CANE in this study was 0.74.

#### The Short Portable Mental Status Questionnaire, SPMSQ

Compared with similar measurement tools of mental status for older adults such as MMSE, the Short Portable Mental Status Questionnaire [16] is simpler and takes less time, which is suitable for screening in older population. The questionnaire has 10 questions and the respondents were scored based on the tally of correct answers. A tally of 0–2 correct answers was deemed as having very severe cognitive impairment, 3–5 as severe cognitive impairment, 6–7 as mild cognitive impairment and 8–10 as having normal cognitive function. Although the Chinese version of SPMSQ has been widely used in older adults, there are few studies exploring the threshold value. A Singapore study with a majority of Chinese participants found that the sensitivity (78%) and specificity (75%) of SPMSQ were better when the threshold value was “5 or fewer correct answers”, and the older population having less than 6 years of education could make 4 correct answers [9]. Therefore, this study specified that older adults making “5 or more fewer correct answers” were deemed as having severe cognitive impairment. The scale has good reliability [17].

#### Demographic and health status questionnaire

The researchers developed the demographic and health status questionnaire by themselves. The demographic items included organizational type, staffing, room type, age, gender, ethnicity, religion, education, marital status, having living children or not, length of time of living in nursing homes (years), living condition before admission, income (yuan/month). The health status items included eyesight, hearing, number of diseases, sleeping status, skin conditions, occurrence of accidents in the past 30 days, all of which were based on participants’ self-report.

#### Modified Barthel Index

The Modified Barthel Index was used to assess 10 activities of daily living (ADL): grooming, bathing, feeding, toilet use, stair climbing, dressing, bladder, bowels, walking or wheelchair movement, and transfer (chair to bed and back) [18]. The total score of the scale was 100, with a score ≤ 40 indicating severely dependent, 45–60 moderately dependent, 65–95 slightly dependent, and 100 completely independent. The Cronbach’s alpha coefficient for the ADL in this study was 0.85.

#### The Numerical Rating Scale (NRS) for pain assessment

The NRS for pain assessment divides pain into 0–10 points: the higher the score is, the more severe the pain is. The NRS for pain assessment has a good acceptance among older population [10]. A score of 0 indicates no pain, 1–3 mild pain, 4–6 moderate pain and 7–10 severe pain. The NRS for pain assessment has been tested by various studies and has good reliability [19].

#### The 15-item Geriatric Depression Scale (GDS-15)

GDS-15 has been widely used in the screening of depressive symptoms of older adults and has good reliability and validity [11]. The 15-item scale assesses how older adults felt over the past week, with “yes” (scoring 1) or “no” (scoring 0) responses. Five of the questions used reverse scoring. The score ranges from 0 to 15, with higher scores suggesting more severe depression. A systematic review has shown that the Chinese version of the scale for older adults takes 4 or 5 points as the cut-off point mostly [11]. In this study, we used a score of 5 or above to specify the existence of depression, in an effort to facilitate the comparison between similar studies. The Cronbach’s alpha coefficient for the GDS-15 in this study was 0.80.

### Data analysis

Epidata 3.1 was used for data entry. SPSS 25.0 was adopted for statistical analysis. The counting data were described by frequency and percentages; the measurement data were examined for normality with Shapiro-Wilk test, with normally distributed data being described by mean ± standard deviation, and non- normally distributed data being described by median (interquartile range, abbreviated as *IQR*).

Univariate Logistic regression analysis was used to test whether there were differences in unmet needs among research participants with different characteristics. Hierarchical logistic regression analysis was then employed to analyze the influencing factors of unmet needs of the elderly nursing home residents. The criteria for including and excluding variables were α_in_ = 0.05 and α_out_ = 0.10.

## Results

### Results of demographic data, health status and covariates of older adults

The average age of the 2063 nursing homes residents in this study was 82.15 ± 6.80 (median: 83; IQR: 79, 86). Among them, 1308 were female (63.4%) and 755 were male (36.6%). Per capita income was 4158.34 ± 1929.82 (median: 4000, IQR:3000, 5000). Average length of stay of the participants was 36.59 ± 37.54 months (median: 24; IQR: 8, 51). Among the participants, 42.1% reported abnormal eyesight, 25.7% abnormal hearing, 42.9% abnormal sleep status, and 21.2% abnormal skin status; 61.3% had 1–2 diseases, and 25.8% had 3 or more diseases. Demographic data and health status of the older adults in nursing homes were shown in Table 1.

**Table 1:** Results of demographic data, health status and covariates of older adults in nursing homes

Variables	Category of variables	n (%)
Age (years old)	60–69	112(5.4)
	70–79	438(21.2)
	80–89	1293(62.7)
	≥ 90	216(10.5)
Gender	Male	755(36.6)
	Female	1308(63.4)
Institution type	Privately operated	849(41.2)
	Publicly operated	1214(58.8)
Ethnicity	Han	2025(98.2)
	Other	38(1.8)
Religion	None	1779(86.2)
	Have	282(13.7)
Educational background	Primary school and below	758(36.7)
	Middle school/high school/secondary school	869(42.1)
	Junior college/university and over	428(20.7)
Marital status	Married	640(31.0)
	Other (widowed/divorced)	1417(68.7)
Having children or not	Have	2001(97.0)
	None	60(2.9)
Length of time of living in nursing homes (year)	≤ 1	504(24.4)
	1–3	514(24.9)
	3–5	313(15.2)
	≥ 5	314(15.2)
Living condition before admission	Solitude	634(30.7)
	Living with spouse	849(41.2)
	Living with children	489(23.7)
	Other	89(4.3)
Room type	Single room	497(24.1)
	Twin room	1314(63.7)
	Dormitory	236(11.4)
Income (yuan/month)	≤ 1999	100(4.8)
	2000–4999	1096(53.1)
	≥ 5000	585(28.4)
Eyesight	Normal	1192(57.8)
	Abnormal	869(42.1)
Hearing	Normal	1526(74.0)
	Abnormal	531(25.7)
Number of diseases	0	248(12.0)
	1–2	1265(61.3)
	≥ 3	533(25.8)
Sleep status	Normal	1177(57.1)
	Abnormal	886(42.9)
Skin status	Normal	1626(78.8)
	Abnormal	437(21.2)
Occurrence of accidents in the past 30 days	None	1826(88.5)
	Have	227(11.0)
Staffing	Enough	1037(50.3)
	Relatively enough	411(19.9)
	Ordinary	279(13.5)
	Relatively inadequate	220(10.7)
	Very inadequate	101(4.9)
Pain	None	1116(54.1)
	Slightly	702(34.0)
	Moderate	192(9.3)
	Severe	49(2.4)
ADL scoring	Independent	840(40.7)
	Mildly dependent	1080(52.4)
	Moderately/severely dependent	32(1.6)
Depression	None	1654(80.2)
	Have	409(19.8)

Pain: The Numerical Rating Scale for pain assessment; ADL: Modified Barthel Index; Depression: Geriatric Depression Scale. Other variables were collected by the Demographic and health status questionnaire

The mean of SPMSQ was 9.38 ± 1.14 (median:10; IQR: 9, 10). The mean score of pain was 1.28 ± 1.89 (median: 0; IQR: 0, 2). Average Barthel Index was 89.58 ± 15.53 (median: 95; IQR: 85, 100). The mean score of GDS-15 was 2.60 ± 2.86 (median: 2; IQR: 0, 4). Categories of the above-mentioned covariates were also shown in Table 1.

### Needs of the older adults in nursing homes

The distribution of the older adults by category of needs for CANE items were shown in Table 2. The tally of no needs for all CANE items was 34,329, accounting for 83.2% of the tally of the four categories (no needs, met needs, unmet needs, and unclear needs). The median and inter-quartile range of the total needs and unmet needs of the sample was 3(1, 4) and 0(0, 1) respectively. Older adults had a higher proportion of met needs in looking after the home (51.1%), food (56.0%), self-care (26.8%), mobility/falls (14.7%), physical health (27.1%). Unmet needs included food (18.3%), daily activity (11.2%), memory (6.0%), physical health (7.6%), information (physical condition & treatment) (13.9%), company (11.9%), and intimate relationships (9.2%).

**Table 2:** The distribution of older adults by category of needs for CANE items

Item	No need n (%)	Met need n (%)	Unmet need n (%)	Unclear n (%)	Total needs n (%)
Look after the home	969(47.0)	1054(51.1)	32(1.6)	2(0.1)	1086(52.8)
Food	526(25.5)	1155(56.0)	378(18.3)	0(0.0)	1533(74.5)
Self-care	1487(72.1)	552(26.8)	22(1.1)	0(0.0)	574(27.9)
Daily activity	1654(80.2)	174(8.4)	232(11.2)	0(0.0)	406(19.7)
Memory	1854(89.9)	86(4.2)	123(6.0)	0(0.0)	209(10.1)
Eyesight/hearing/communication	1921(93.1)	66(3.2)	76(3.7)	0(0.0)	142(6.9)
Mobility/falls	1702(82.5)	304(14.7)	57(2.8)	0(0.0)	361(17.5)
Control	1983(96.1)	72(3.5)	8(0.4)	0(0.0)	80(3.9)
Physical health	1347(65.3)	560(27.1)	156(7.6)	0(0.0)	716(34.7)
Medication	1841(89.2)	158(7.7)	64(3.1)	0(0.0)	222(10.8)
Psychotic symptoms	2047(99.2)	12(0.6)	4(0.2)	0(0.0)	16(0.8)
Psychological distress	1979(95.9)	42(2.0)	42(2.0)	0(0.0)	84(4.1)
Information (physical condition & treatment)	1615(78.3)	160(7.8)	286(13.9)	0(0.0)	446(21.6)
Deliberate self-harm	2053(99.5)	6(0.3)	4(0.2)	0(0.0)	10(0.5)
Inadvertent self-harm	2021(98.0)	30(1.5)	12(0.6)	0(0.0)	42(2.0)
Abuse/neglect	2031(98.4)	24(1.2)	8(0.4)	0(0.0)	32(1.6)
Behavior	2043(99.0)	16(0.8)	4(0.2)	0(0.0)	20(1.0)
Company	1668(80.9)	150(7.3)	245(11.9)	0(0.0)	395(19.1)
Intimate relationships	1759(85.3)	112(5.4)	190(9.2)	0(0.0)	302(14.7)
Benefits	1829(88.7)	162(7.9)	56(2.7)	14(0.7)	218(10.6)
**Total**	34,329(83.2)	4895(11.9)	1999(4.8)	16(0.0)	6894(16.7)

Total needs = the number of older adults with met needs + the number of older adults with unmet needs

*CANE* Camberwell Assessment of Need for the elderly

The tally of unmet needs for all CANE items was 1999, accounting for 4.8% of the tally of the four categories (no needs, met needs, unmet needs, and unclear). Given that the developers of CANE did not specify how large of unmet needs is defined as low or high, plus the preponderance of older adults without a need, we took the 90th percentile of unmet needs as the tentative cut-off point. The unmet needs of the older adults were listed in ascending order, and the 90th percentile was 3. Thus, the older adults with ≤ 3 unmet needs were grouped into the low unmet need category, while those with > 3 needs into the high unmet need category. Results showed that the unmet needs of 1922 residents were ≤ 3 and the unmet needs of 122 residents were > 3.

### Univariate logistic regression analysis of unmet needs of older adults in nursing homes

Univariate logistic regression analysis showed that organization type, age, gender, religion, educational background, marital status, length of time of living in nursing homes (year), living condition before admission, room type, income (yuan/month), staffing, eyesight, hearing, sleeping status, skin status, occurrence of accidents in the past 30 days, pain, ADL scoring and depression were the influencing factors for unmet needs of the residents in nursing homes. The results of univariate logistic regression analysis of the unmet needs of older adults were shown in Table 3.

**Table 3:** Logistic regression analysis results of the influencing factors for the unmet needs of the older adults in nursing homes

Variable	Category of variable	Low unmet needs n(%)	High unmet needs n(%)	Univariate analysis	Hierarchical analysis model 1 model 2 model 3 model 4 model 5				
				OR value (95%CI)	OR value (95%CI)	OR value (95%CI)	OR value (95%CI)	OR value (95%CI)	OR value (95%CI)
Age (years old)	60–69	96 (85.7)	16 (14.3)	Reference	Reference	Reference	Reference	Reference	Reference
	70–79	410 (94.0)	26 (6.0)	**0.38 (0.20,0.74)**	0.60 (0.22,1.70)	0.56 (0.19,1.65)	0.55(0.18,1.68)	4.15(0.37,46.82)	6.18(0.48,79.31)
	80–89	1209 (94.7)	68 (5.3)	**0.34(0.19,0.60)**	0.41 (0.16,1.07)	**0.28 (0.10,0.76)**	**0.30(0.11,0.86)**	2.40(0.22,26.46)	2.97(0.24,36.62)
	≥ 90	203 (94.4)	12 (5.6)	**0.36(0.16,0.78)**	0.49 (0.14,1.74)	0.39(0.10,1.47)	0.35(0.09,1.40)	2.09(0.16,27.64)	2.51(0.17,37.94)
Gender	Male	691 (92.0)	60 (8.0)	Reference	Reference	Reference	Reference	Reference	Reference
	Female	1231 (95.2)	62 (4.8)	**0.58 (0.40,0.84)**	**0.34 (0.19,0.60)**	**0.31(0.17,0.58)**	**0.24(0.13,0.46)**	**0.26(0.12,0.57)**	**0.33(0.14,0.79)**
Institution type	Private operated	761 (90.9)	76 (9.1)	Reference	Reference	Reference	Reference	Reference	Reference
	Public operate	1161 (96.2)	46 (3.8)	**0.40 (0.27,0.58)**	0.60 (0.34,1.06)	0.60 (0.33,1.12)	0.71(0.38,1.33)	1.02(0.45,2.27)	1.17(0.51,2.68)
Ethnicity	Han	1886 (94.0)	120 (6.0)	Reference	Reference	Reference	Reference	Reference	Reference
	Other	36 (94.7)	2 (5.3)	0.87 (0.21,3.67)	0.00 (0.00, -)	0.00(0.00,-)	0.00(0.00,-)	0.00(0.00,-)	0.00(0.00,-)
Religion	None	1666 (94.4)	98 (5.6)	Reference	Reference	Reference	Reference	Reference	Reference
	Have	256 (91.4)	24 (8.6)	**1.59 (1.00,2.54)**	**2.32 (1.11,4.86)**	**2.82(1.29,6.16)**	**3.62(1.61,8.17)**	2.46(0.85,7.14)	**3.21(1.07,9.68)**
Educational background	Primary school and below	675 (90.4)	72 (9.6)	Reference	Reference	Reference	Reference	Reference	Reference
	Middle school/high school/secondary school	825 (95.6)	38 (4.4)	**0.43 (0.29,0.65)**	**0.34 (0.19,0.62)**	**0.33(0.17,0.62)**	**0.33(0.17,0.65)**	**0.22(0.09,0.54)**	**0.25(0.10,0.62)**
	Junior college/university and over	414 (97.2)	12 (2.8)	**0.27 (0.15,0.51)**	**0.27 (0.10,0.73)**	0.37(0.13,1.03)	0.48(0.17,1.36)	0.96(0.30,3.09)	1.07(0.32,3.54)
Marital status	Married	680 (96.3)	26 (3.7)	Reference	Reference	Reference	Reference	Reference	Reference
	Other (widowed/divorced)	1230 (92.8)	96 (7.2)	**0.49 (0.31,0.76)**	0.61 (0.29,1.30)	0.60(0.27,1.33)	0.46(0.20,1.06)	**0.34(0.12,0.98)**	**0.21(0.07,0.69)**
Having children or not	Have	1864 (94.0)	118 (6.0)	Reference	Reference	Reference	Reference	Reference	Reference
	none	56 (93.3)	4 (6.7)	1.13 (0.40,3.17)	0.86 (0.15,4.78)	0.60(0.09,3.98)	0.42(0.05,3.91)	0.98(0.08,12.56)	1.45(0.09,24.61)
Length of time of living in nursing homes (year)	≤ 1	470 (94.0)	30 (6.0)	Reference	Reference	Reference	Reference	Reference	Reference
	1 ~ 3	478 (94.1)	30 (5.9)	0.98 (0.58, 1.66)	1.31 (0.64,2.70)	1.20(0.57,2.51)	1.31(0.61,2.82)	0.98(0.39,2.50)	1.01(0.38,2.69)
	3 ~ 5	293 (94.8)	16 (5.2)	0.86 (0.46,1.60)	1.58 (0.69,3.62)	1.45(0.61,3.46)	1.77(0.72,4.34)	0.84(0.27,2.64)	0.94(0.29,2.99)
	≥ 5	274 (88.4)	36 (11.6)	**2.06 (1.24,3.42)**	2.11 (0.95,4.68)	1.66(0.72,3.84)	1.66(0.69,4.00)	0.89(0.30,2.60)	1.08(0.36,3.25)
Living condition before admission	Solitude	585 (93.0)	44 (7.0)	Reference	Reference	Reference	Reference	Reference	Reference
	Living with spouse	783 (93.1)	58 (6.9)	0.99 (0.66,1.48)	**2.64 (1.34,5.23)**	**2.35(1.13,4.91)**	**2.27(1.05,4.90)**	2.28(0.88,5.87)	**3.05(1.11,8.33)**
	Living with children	463 (95.9)	20 (4.1)	**0.57 (0.33,0.99)**	0.77 (0.35,1.72)	0.73(0.31,1.70)	0.82(0.35,1.92)	0.50(0.16,1.59)	0.54(0.16,1.83)
	Other	89 (100.0)	0 (0.0)	0.00 (0.00, -)	0.00 (0.00, -)	0.00(0.00,-)	0.00(0.00,-)	0.00(0.00,-)	0.00(0.00,-)
Room type	Single room	429 (87.4)	62 (12.6)	Reference	Reference	Reference	Reference	Reference	Reference
	Twin room	1261 (96.8)	46 (3.2)	**0.23 (0.15,0.35)**	**0.43 (0.23,0.78)**	**0.43(0.23,0.82)**	**0.39(0.20,0.75)**	**0.38(0.17,0.85)**	**0.43(0.19,0.98)**
	Dormitory	222 (94.1)	14 (5.9)	**0.44 (0.24,0.80)**	0.51 (0.21,1.24)	**0.35(0.14,0.88)**	**0.31(0.12,0.80)**	0.00(0.00,-)	0.00(0.00,-)
Income (yuan/month)	≤ 1999	72 (73.5)	26 (26.5)	Reference	Reference	Reference	Reference	Reference	Reference
	2000 ~ 4999	1035 (95.0)	54 (5.0)	**0.14 (0.09,0.24)**	**0.14 (0.07,0.29)**	**0.15(0.07,0.33)**	**0.18(0.08,0.42)**	**0.11(0.04,0.34)**	**0.10(0.03,0.33)**
	≥ 5000	567 (97.9)	12 (2.1)	**0.06 (0.03,0.12)**	**0.06 (0.02,0.16)**	**0.05(0.01,0.14)**	**0.06(0.02,0.18)**	**0.02(0.01,0.11)**	**0.03(0.01,0.15)**
Staffing	Enough	987 (96.1)	40 (3.9)	Reference	Reference	Reference	Reference	Reference	Reference
	Relatively enough	375 (92.1)	32 (7.9)	**2.11 (1.30,3.40)**	**2.24 (1.12,4.48)**	**2.67(1.28,5.56)**	**2.94(1.33.6.49)**	**2.81(1.07,7.36)**	2.66(0.96,7.38)
	Ordinary	253 (91.3)	24 (8.7)	**2.34 (1.39,3.96)**	**3.26 (1.43,7.44)**	**4.32(1.78,10.52)**	**5.11(2.10,12.44)**	**6.45(2.01,20.71)**	**6.79(2.02,22.82)**
	Relatively inadequate	204 (92.7)	16 (7.3)	**1.94 (1.06,3.52)**	**3.25 (1.39,7.62)**	**3.14(1.26,7.81)**	**4.46(1.69,11.74)**	**8.08(2.49,26.21)**	**7.63(2.26,25.76)**
	Very inadequate	89 (89.9)	10 (10.1)	**2.77 (1.34,5.73)**	**4.59 (1.33,15.87)**	**4.18(1.10,15.83)**	2.70(0.66,11.12)	3.58(0.64,20.00)	2.28(0.35,15.02)
Eyesight	Normal	1138 (96.4)	42 (3.6)	Reference		Reference	Reference	Reference	Reference
	Abnormal	782 (90.7)	80 (9.3)	**2.77 (1.89,4.07)**		1.58(0.88,2.86)	1.66(0.90,3.06)	1.90(0.91,3.95)	1.87(0.85,4.12)
Hearing	Normal	1443 (95.4)	70 (4.6)	Reference		Reference	Reference	Reference	Reference
	Abnormal	473 (90.1)	52 (9.9)	**2.27 (1.56,3.29)**		**2.65(1.49,4.72)**	**2.82(1.55,5.14)**	**2.15(1.02,4.56)**	2.21(0.99,4.96)
Number of diseases	0	236 (95.2)	12 (4.8)	Reference		Reference	Reference	Reference	Reference
	1 ~ 2	1181 (94.6)	68 (5.4)	1.13 (0.60,2.13)		**3.37(1.09,10.43)**	**3.58(1.09,11.76)**	**10.68(1.89,60.49)**	**12.40(2.09,73.38)**
	≥ 3	488 (92.1)	42 (7.9)	1.69 (0.88,3.28)		**3.42(1.02,11.43)**	3.60(0.99,13.05)	**10.47(1.62,67.82)**	**10.95(1.57,76.54)**
Sleeping status	Normal	1115 (95.9)	48 (4.1)	Reference		Reference	Reference	Reference	Reference
	Abnormal	807 (91.6)	74 (8.4)	**2.13 (1.47,3.10)**		**1.81(1.01,3.21)**	**1.88(1.03,3.43)**	1.62(0.77,3.42)	1.78(0.82,3.85)
Skin status	Normal	1529 (94.7)	86 (5.3)	Reference		Reference	Reference	Reference	Reference
	Abnormal	393 (91.6)	36 (8.4)	**1.63 (1.09,2.44)**		1.73(0.90,3.33)	1.70(0.86,3.36)	1.29(0.54,3.08)	1.17(0.47,2.96)
Occurrence of accidents in the past 30 days	None	1715 (94.7)	96 (5.3)	Reference		Reference	Reference	Reference	Reference
	Have	201 (88.5)	26 (11.5)	**2.31 (1.46,3.65)**		1.62(0.68,3.83)	0.79(0.30,2.10)	0.44(0.10,1.88)	0.27(0.06,1.27)
Pain	None	1058 (95.5)	50 (4.5)	Reference			Reference	Reference	Reference
	Slightly	661 (95.1)	34 (4.9)	1.09 (0.70,1.70)			1.21(0.61,2.39)	0.94(0.41,2.14)	0.96(0.41,2.26)
	Moderate	160 (84.2)	30 (15.8)	**3.97 (2.45,6.43)**			**4.04(1.64,9.94)**	3.10(0.94,10.18)	2.77(0.76,10.06)
	Severe	41 (83.7)	8 (16.3)	**4.13 (1.84,9.27)**			**17.52(4.61,66.59)**	**23.00(4.01,131.95)**	**14.98(2.27,99.01)**
ADL levels	Independent	810 (97.1)	24 (2.9)	Reference				Reference	Reference
	Mildly dependent	1007 (94.4)	60 (5.6)	**2.01 (1.24,3.26)**				1.26(0.56,2.83)	0.81(0.34,1.95)
	Moderately/severely dependent	26 (81.3)	6 (18.8)	**7.79 (2.93,20.67)**				**56.08(5.46,576.00)**	**30.22(2.42,377.26)**
Depression	None	1594 (97.4)	42 (2.6)	Reference					Reference
	Have	328 (80.4)	80 (19.6)	**9.26 (6.26,13.70)**					**6.76(2.88,15.85)**
	R^2^ of Negorko				0.306	0.373	0.411	0.460	0.503
	R^2^ of Cox-Snell				0.109	0.135	0.148	0.144	0.158
	*P*				0.000	0.000	0.000	0.000	0.000

Model 1, adjusted for demographic characteristics (institution type, age, gender, ethnicity, religion, educational background, marital status, having children or not, length of time of living in nursing homes, living condition before admission, room type, income, staffing) ; Model 2, adjusted for demographic characteristics + health status; model 3, adjusted for demographic characteristics + health status + pain; model 4, adjusted for demographic characteristics + health status + pain + ADL; model 5, adjusted for demographic characteristics + health status + pain + ADL + depression. Pain: The Numerical Rating Scale for pain assessment; ADL: Modified Barthel Index; Depression: Geriatric Depression Scale; Unmet needs: older adults with ≤ 3 unmet needs were grouped into the low unmet need category(coded 0), while those with > 3 needs into the high unmet need category (coded 1)

### Hierarchical logistic regression analysis of unmet needs of the residents in nursing homes

The independent variables were divided into five levels: (1) demographic characteristics (institution type, age, gender, ethnicity, religion, educational background, marital status, having children or not, length of time of living in nursing homes, living condition before admission, room type, income, staffing), (2) health status (eyesight, hearing, number of diseases, sleeping status, skin status, accidents in the past 30 days), (3) pain, (4) self-care ability (namely ADL) and (5) depression, and the dependent variable was the degree of unmet needs ( high *versus* low). Hierarchical logistic regression analysis was performed by controlling for the 5 levels of independent variables, yielding 5 models.

The results of hierarchical logistic regression analysis showed that the R^2^ of Negorko increased with each additional level of independent variables and was statistically significant.

Hierarchical logistic regression analysis results showed that the *P* value of Omnibus test of the final model was < 0.001, suggesting that the model turned out well. The *P* value of Hosmer-Lemshaw test of the final model was 0.073 > 0.05, implying that the model fitted well. The value of Cox-Snell’s R^2^ stood at 0.158, versus a Negorko’s R^2^ at 0.503. Gender, religion, educational background, marital status, living condition before admission, room type, incomes (yuan/month), staffing, number of diseases, pain, Barthel Index, and depression were the influencing factors for the unmet needs of older adults in nursing homes, which could be seen in Table 3.

## Discussion

In our study, the tally of no needs for all CANE items accounted for the largest share of the four categories, followed by tally of met needs. From these facts we can infer that that most of the needs were addressed. The tally of unmet needs for all CANE items in older adults was far lower than the tallies of no needs and met needs. This result was consistent with previous studies conducted in residential care [6, 20]. A lower number of unmet needs might also indicate that older adults were receiving adequate care and services from nursing homes. The proportion of the unmet needs category in the study was lower than the finding by Ferreira et al. [21], which could be explained by the differences in participants’ selection – our study only included older adults without severe cognitive disorders whereas the latter included 58.7% of the older adults with cognitive deficit. The proportion of the unmet needs category in our study was also lower than that in primary care [15], community dwelling older adults with dementia [22], depressed primary care older patients [23], and the oldest old primary care patients with common somatic and psychiatric disorders [5]. The difference in the results might be attributable to variation in study design, sample selection and methodology.

Our study casts spotlight on seven aspects of unmet needs: food, daily activity, memory, physical health, information (physical condition & treatment), company, and intimate relationships. Most unmet needs of the residents fell into social domain, which is in line with the research findings by van den Brink et al. [7], Ferreira et al. [21] and Tobis et al. [6, 8]. This implied that nursing homes may focus on the daily care for older adults and neglect the social needs of them. Research reported that stronger social connections were associated with better mental health outcomes [24]. Launching reminiscence therapy and organizing mutual support groups for older adults have been tested effective to meet the social needs of them [25] and are suggested. Food was among the most prevalent unmet needs in this population, which was inconsistent with previous studies [7, 8, 21]. The possible reason is that some older adults had bad teeth, diabetes, hypertension, dysphagia, etc., or had their own special eating habits and they needed specific diets or staffer’s assistance. However, the simple and repeated food supply, lack of staffer’s assistance in nursing homes could not meet the needs of this part of older population [26]. Promoting food diversity according to the dietary characteristics of older adults and assisting those with difficult food intake by nursing home staff could be effective ways to solve this problem.

That the need for physical health was unmet reflected inadequate provision of healthcare services in some residential institutions. China has been promoting the strategy of “Integrated Medical and Geriatric Care Services” as a solution to meet the physical care needs of older adults since 2016 [27]. Our finding highlights that this policy should be further rolled out in more nursing homes. What’s more, memory was found to be one of the prevalent unmet needs among older adults in care homes as well, supported by Ferreira et al. [21] and Iliffe et al. [28].

The result of hierarchical logistic regression analysis in this study showed that gender was an independent influencing factors for the unmet needs of older adults in nursing homes, with males having higher odds of falling into the high unmet need category than females in all the five regression models. Result of oldest old in nursing homes from rural areas by Zhu supported our finding [29]. This could be explained by the traditional sociocultural pattern of women as caregivers and men as employees in society [30] where women had lower expectations for care services and thus were more likely to show satisfaction with the care they received [29]. The finding was inconsistent with the research results of Tobis et al., who found that there was no gender difference of unmet needs between female and male residents aged over 75 years [8]. It can be inferred that the unmet needs of older adults in different age groups showed disparities.

Whether older adults in nursing homes had religious beliefs influenced their unmet needs level in almost all the regression models, which indicated that older adults with religious beliefs reported higher level of unmet needs than those without. The reason might be that at present, nursing homes in mainland China are short on religious amenities or services to meet the needs of residents with religious beliefs [31]. Our results highlighted religious support by caregivers as an essential measure to meet spiritual needs of nursing home residents.

Our study found that in the first regression model, the higher the education attainment, the lower the extent of unmet needs. This finding was in line with the research by Liu et al. [32]. Older persons with higher education level tend to have higher incomes and better access to nursing home services. However, in subsequent regression models,

compared with the residents having an educational background of primary school and below, those who joined junior college or above showed no significant difference in the extent of unmet needs. It can be inferred that health status, pain, self-care ability and depression may play certain mediating role in the association between personal characteristics and unmet needs among those who had the highest educational level. The mediating effect of these variables among this population needs to be further tested.

Older adults who were widowed or divorced had lower odds of falling into the high unmet need category than those who had spouses. The association was insignificant in model 1 through model 3, but became otherwise significant after controlling for ADL (model 4) and depression (model 5). This is in echoes with our another finding that older adults who had been living with their spouse before admission had higher odds of falling into the high unmet need category than those who had been living in solitude or living with children. That having a living spouse is predictive of higher extent of unmet need is worth exploring. Study showed that spouses, hailed as an important source of care and comfort in many settings, could meet the needs of older adults by providing company and care, thus reducing mutual loneliness and promoting overall health [33]. However, for older residents living apart from his/her spouse, especially for those once living with their spouse before admission, they may find the care they received from nursing homes is nowhere on a par with what they received from spouses [34]. For older individuals who have been living in solitude, they tend not to have the care gap. Hence, they had lower unmet needs. Our result is inconsistent with the findings of other studies [4, 35], which might be explained by disparities in older populations residing in various settings, or disparities in proportions of older residents who were separated from their spouses.

All the regression models in this study showed that compared with the residents living alone, those living with one roommate had lower level of unmet needs in nursing homes. Study implied that roommate could help around and offer emotional support for each other, which is beneficial to residents’ psychological health and quality of life [36]. Helping older adults build good connections with their roommates could be a solution to meet the social needs of them in long term care. However, for those living with more than one roommate, the result of this study was complex and difficult to interpret. More evidence is needed to consolidate the finding.

Higher incomes indicated fewer unmet needs among nursing home residents in all the regression models, which was in accordance with the study by Liu et al. [32]. The reason might be that older adults with higher incomes tend to opt for nursing homes which provide better services, thus having fewer unmet needs. Plus, richer older adults may have healthier life styles as well [37].

Shortage of human resources was another independent facilitating factor for the unmet needs of older adults in nursing homes in almost all the regression models. Insufficient personnel allocation cannot timely meet the needs of clients, which will greatly affect the level of services, the health outcomes of the residents and the development of the institutions [38]. In theory, older adults who rated staffing as very inadequate are more likely to fall into the high unmet need category than those who rated staffing as enough. But our results contradict the theory. From the fact that the older adults who rated staffing as very inadequate made up a small share in our study, we can infer that small sample size of this group may bring deviation to regression models, which is in need of further verification in future studies.

This study showed that abnormal eyesight and hearing were not facilitators for older adults’ membership into the high unmet needs category in the final model. This was inconsistent with findings from Pittman, et al. [39]. Our study also failed to prove abnormal sleeping or skin status contributive to high level of unmet needs among older adults. One possible explanation is that older adults may believe that changes in eyesight, hearing, sleep patterns and skin conditions come naturally and inevitably with aging and should be made light of when these changes have not incurred too many troubles to their daily life. To our knowledge, there is hardly any evidence regarding the relationship between sleeping status as well as skin status and the level of unmet needs. These findings need to be further tested.

Compared with older adults with no pain, those with severe pain were likely to have higher extent of unmet needs. This result supported the evidence that some nursing homes fail to meet the needs of older adults with severe pain [40]. Nursing home administrators could ramp up their cooperation with hospitals in pain assessment and management for older adults. Likewise, being moderately or severely dependent facilitated older adults’ membership into the high unmet needs category. These results could be explained by that nursing home staff lack the competencies, time and energy to meet the needs of residents who were moderately or severely dependent [41]. The government should scale up support for nursing homes and train more high quality staff to meet the needs of these older adults.

Older adults with depression in nursing homes tended to have higher extent of unmet needs. This is consistent with previous studies [42–44], indicating that the current long - term care institutions come short of effective measures to alleviate residents’ depressive symptoms. A wide range of environmental, psychological and social factors were related to depression [44], which calls for a raft of collective efforts to mitigate depressive symptoms. This also explains the high extent of unmet needs of depressive residents, as nursing homes, especially those predominantly providing life-assisted care, may be unable to provide multifarious care to meet the needs of depressive residents. Our study highlights the necessity of providing depression screening and collective, manifold care to nursing home residents with depressive symptoms.

### Strengths and limitations

The data of this study were of high quality depending on its strict study design. Due to the limitations of cross-sectional design, we could not draw a causal conclusion of these variables. Plus, only older adults with normal cognitive function or mild cognitive impairment were included in the study and those with severe cognitive impairment were excluded as they were unable to comprehend survey contents. This might result in a bulk of residents with unmet needs being left out in the survey, thus underestimating the scale and extent of unmet needs of nursing home residents. What’s more, due to our failure to recognize the contextual effect possibly exerted by uneven regional economic development, the demographic questionnaire we designed did not include information about regions and cities where the nursing homes were located, making us unable to perform multi-level modeling to probe into the clustering and contextual effect at regional level. This might affect the robustness of our data analysis.

## Conclusion

The unmet needs of older adults in nursing homes mainly focused on social domains, and are attributable to a plethora of factors. This finding could provide clues for stakeholders to address the issue of unmet needs for this population. System-level support to nursing homes and training of staff, which could arm nursing homes with more resources and help staff build up more competences to respond to residents’ unmet needs, are highlighted. Plus, taking measures to beef up social connections for the older adults to meet their social needs is suggested.

## Data Availability

The datasets generated and analyzed during the current study are not publicly available due to them containing information that could compromise research participant privacy, but are available from the corresponding author on reasonable request.

## Abbreviations

CANE: Camberwell Assessment of Need for the Elderly
NRS for pain assessment: The Numerical Rating Scale for pain assessment
GDS-15: the 15-item Geriatric Depression Scale
SPMSQ: The Short Portable Mental Status Questionnaire
ADL: Activities of Daily Living, Modified Barthel Index
I-CVI: Item-level content validity index
S-CVI: Scale-level content validity index
